# Supplementary material for: Posterior basolateral amygdala to ventral hippocampal CA1 drives approach behaviour to exert an anxiolytic effect
Source: Nat Commun. 2020 Jan 10;11:183. doi: 10.1038/s41467-019-13919-3 (PMC6954243; doi:10.1038/s41467-019-13919-3)
Supplement: Supplementary file 1 — Supplementary Information [file 41467_2019_13919_MOESM1_ESM.pdf]

SUPPLEMENTARY INFORMATION

Posterior basolateral amygdala to ventral hippocampal CA1 drives approach behaviour to exert an anxiolytic effect

Pi et al.

Supplementary Figure 1

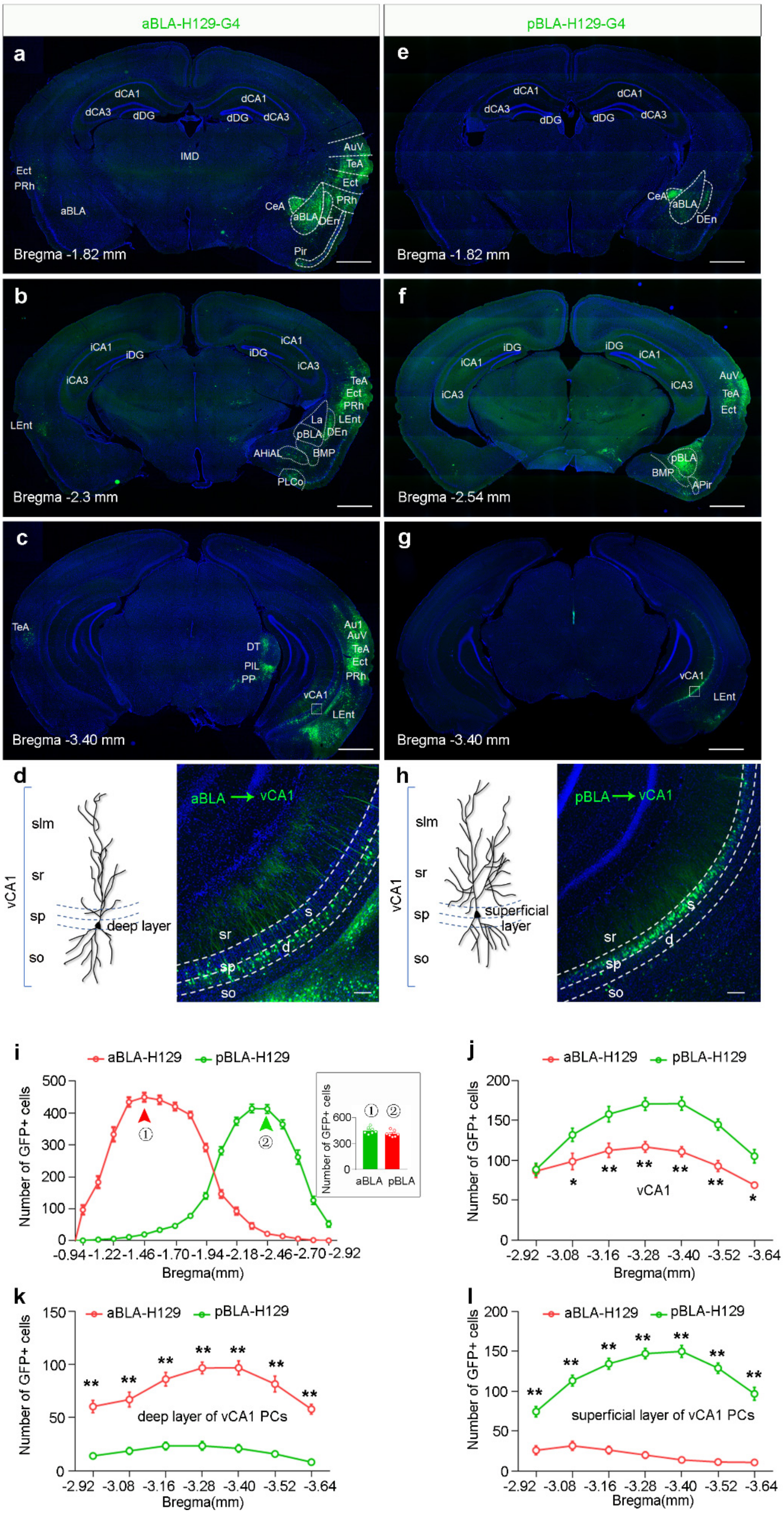

Supplementary Figure 1. Trans–synaptic virus tracing a/pBLA–vCA1 circuits. (**a-g**) Anterograde H129-G4 was injected into the aBLA (**a**) and pBLA (**f**), respectively. 24 h later, mice were sacrificed for series slicing. Representative images of aBLA–(**a-c**) and pBLA-projecting (**e-g**) regions by serial coronal sections. Scale bar=1 mm. (**d, h**) Higher-resolution images of the boxed regions in panel **c** and **g**, showing that H129-G4 injected in aBLA preferentially label the deep layer of vCA1 PCs (**d**), while in pBLA predominantly label the superficial layer of vCA1 PCs (**h**). Scale bar=100 μm. (**i-l**) Comparison of the projection strength of aBLA and pBLA to vCA1: (**i**) Quantitative analysis of the starter cells from anterior (A) to posterior (P) axis in aBLA-H129 and pBLA-H129 mice, respectively. Note that the maximum values of the two groups (aBLA–H129 group at bregma –1.46 vs. pBLA-H129 group at bregma –2.46) was identical. n=7 mice per group, unpaired t test, t=1.842 df=11.99, P=0.0903. (**j**) Comparison of the number of H129-labled vCA1 cells in aBLA-H129 and pBLA-H129 mice, showing that more vCA1 neurons were innervated by pBLA than aBLA from bregma –3.08 to –3.64. n=7 per group, two-way ANOVA, $F_{(6, 72)} = 5.103$, P=0.0002, Sidak's multiple comparisons test, *$P < 0.05$, **$P < 0.01$ vs. pBLA-H129. (**k, l**) Comparison of the number of deep (**k**) and superficial (**l**) vCA1 cells labeled by H129 in aBLA-H129 and pBLA-H129 mice. From bregma –2.92 to –3.64, more H129-labled cells were located in the deep layer of vCA1 PCs in aBLA-H129 mice as compared with those in pBLA-H129 mice (**k**). While the number of H129-labled superficial vCA1 cells was greater in pBLA-H129 mice than those in aBLA-H129 mice (**l**). n=7 mice per group, two-way ANOVA, deep layer of vCA1 PCs: $F_{(6, 72)} = 6.96$, P<0.0001; superficial layer of vCA1 PCs: $F_{(6, 72)} = 22.41$, P<0.0001, Sidak's multiple comparisons test, **$P < 0.01$ vs. pBLA-H129 (**k**) or aBLA-H129 (**l**). so, stratum oriens; sp, stratum pyramidale; sr, stratum radiatum. Source data are provided as a Source Data file.

Supplementary Figure 2

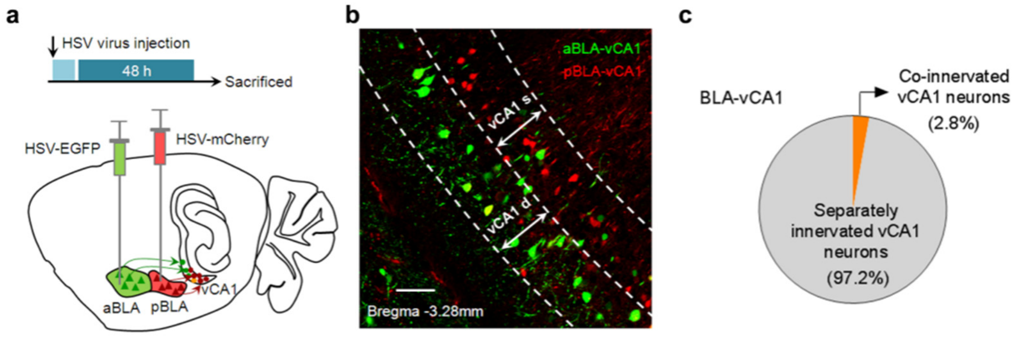

Supplementary Figure 2. Distinct distribution of a/pBLA-innervated vCA1 neurons.

(**a**) Schematic of anterograde multisynaptic tracing to label ventral hippocampal CA1 (vCA1) neurons innervated by aBLA and pBLA. H129-G4 and H129-R4 were injected into aBLA and pBLA respectively. 48 h later, mice were sacrificed to detect eGFP and mCherry in the vCA1. (**b**) The representative image of aBLA- and pBLA-innervated neurons in the vCA1. The aBLA-innervated neurons (eGFP) were predominantly in the deep layer, while the pBLA-innervated neurons (mCherry) in the superficial layer of vCA1. Scale bar, 50 µm. (**c**) Pie chart showing separative and overlapping proportion of aBLA- and pBLA-vCA1 neurons. *n*= 7 mice. Source data are provided as a Source Data file.

Supplementary Figure 3

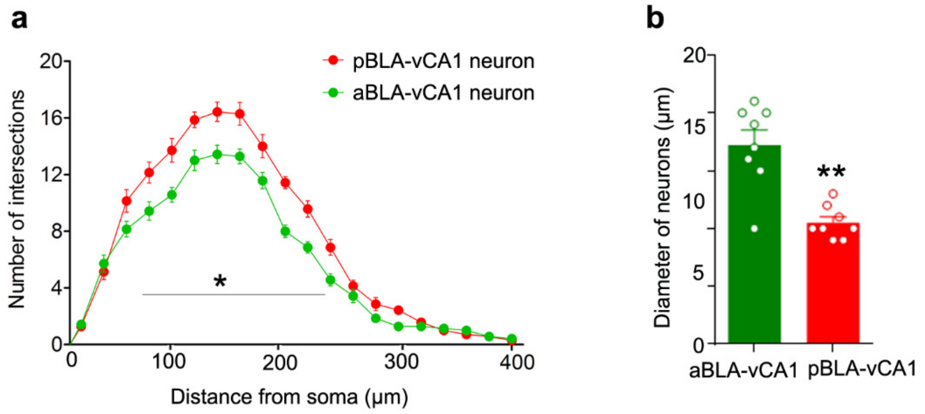

Supplementary Figure 3. Morphological heterogeneity of aBLA- and pBLA-innervated vCA1 neurons.

(**a, b**) Representative images and Sholl analysis shown dendritic complexity. The pBLA–vCA1 neurons are much complicated than the neurons of aBLA–vCA1 (n=7 neurons from 5 mice per group. Two-way ANOVA, Sidak's multiple comparisons test, $*P < 0.05$). (**c**) Diameters of aBLA–vCA1 neurons body were greater than those of pBLA–vCA1 neurons. n=8 neurons from 5 mice per group, unpaired t test, t=4.918 df=14 ,$**P < 0.01$. Data were presented as mean ± SEM. Source data are provided as a Source Data file.

Supplementary Figure 4

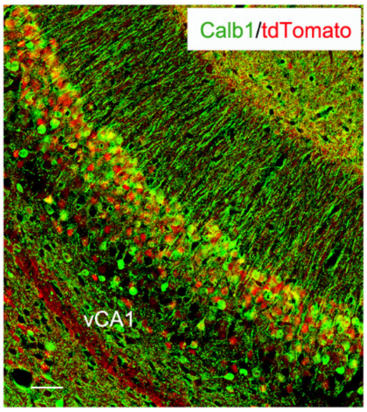

Supplementary Figure 4 Characterization of Calb1-IRES2-Cre-D::Ai9 mice. Example images showing the genetically-tdTomato labeled cells (red) stained with antibody against Calb1 (green) in the vCA1 of Calb1-IRES2-Cre-D::Ai9 mice. Scale bar, 50 μm.

Supplementary Figure 5

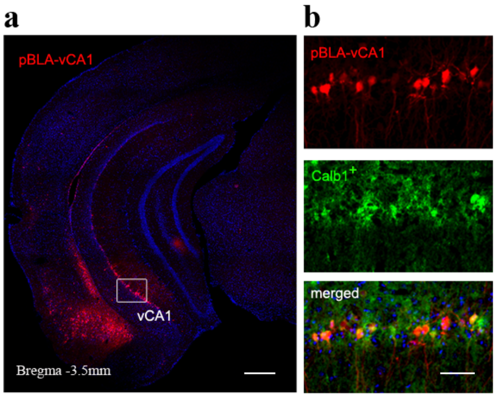

Supplementary Figure 5. Co–localization of pBLA–innervated vCA1 neurons with Calbindin1 (Calb1).

(**a**) Representative image of pBLA-innervated vCA1 neurons by anterograde monosynaptic tracing. Experimental protocol was same as Figure1a. Scale bar, 500 μm.

(**b**) pBLA-innervated vCA1 neurons were probed by co-staining of tdTomato (red) and anti-Calb1 (green). Scale bar, 50 μm.

Supplementary Figure 6

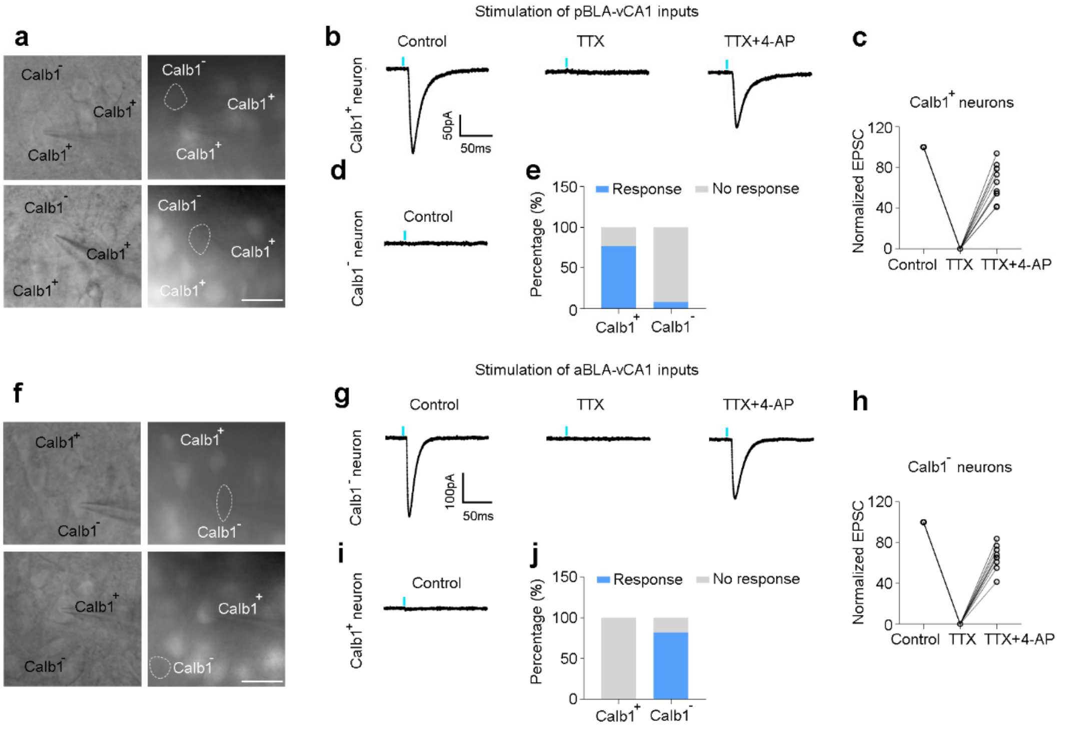

Supplementary Figure 6. Identification of monosynaptic a/pBLA-vCA1 connections. In Calb1-IRES2-Cre-D::Ai9 mice, AAV5-CaMKIIa-hChR2(H134R)-EYFP was injected into the aBLA and pBLA respectively. Four weeks later, electrophysiological response was recorded from vCA1^{Calb1+} and vCA1^{Calb1-} neurons upon the photoactivation of pBLA–vCA1 inputs or aBLA–vCA1 inputs. (**a**, **f**) Representative images of patch pipette tips on vCA1^{Calb1+} (tdTomato+) and vCA1^{Calb1-} (tdTomato-) pyramidal neurons in hippocampal slices (scale bar=30 μm). (**b**, **d, g, i**) Representative traces of EPSCs in the pBLA–vCA1^{Calb1+} pathway (**b**, **d**) and aBLA–vCA1^{Calb1-} pathway (**g, i**) recorded under different experimental conditions. EPSCs were evoked by photostimulation of ChR2-expressing axons from the pBLA and aBLA, and recorded in Calb1^{+} and Calb1^{-} neurons respectively in the vCA1. Optogenetically-induced and tetrodotoxin (TTX)-blocked EPSCs were partially rescued by 4-aminopyridine (4-AP), indicating monosynaptic nature of connections in the pBLA to the vCA1^{Calb1+} and aBLA to the vCA1^{Calb1-} pathways. (**c**, **h**) Changes of EPSCs amplitude in TTX only and TTX + 4-AP. (**e, j**) Percentage of vCA1^{Calb1+} and vCA1^{Calb1-} neurons in response to the photostimulation of pBLA–vCA1 (**e**) and aBLA–vCA1 pathways (**j**). n=13 vCA1^{Calb1+} neurons and 12 vCA1^{Calb1-} neurons from 10 pBLA-vCA1-ChR2 mice; n=11 vCA1^{Calb1+} and 11 vCA1^{Calb1-} neurons from 9 aBLA-vCA1-ChR2 mice. Source data are provided as a Source Data file.

Supplementary Figure 7

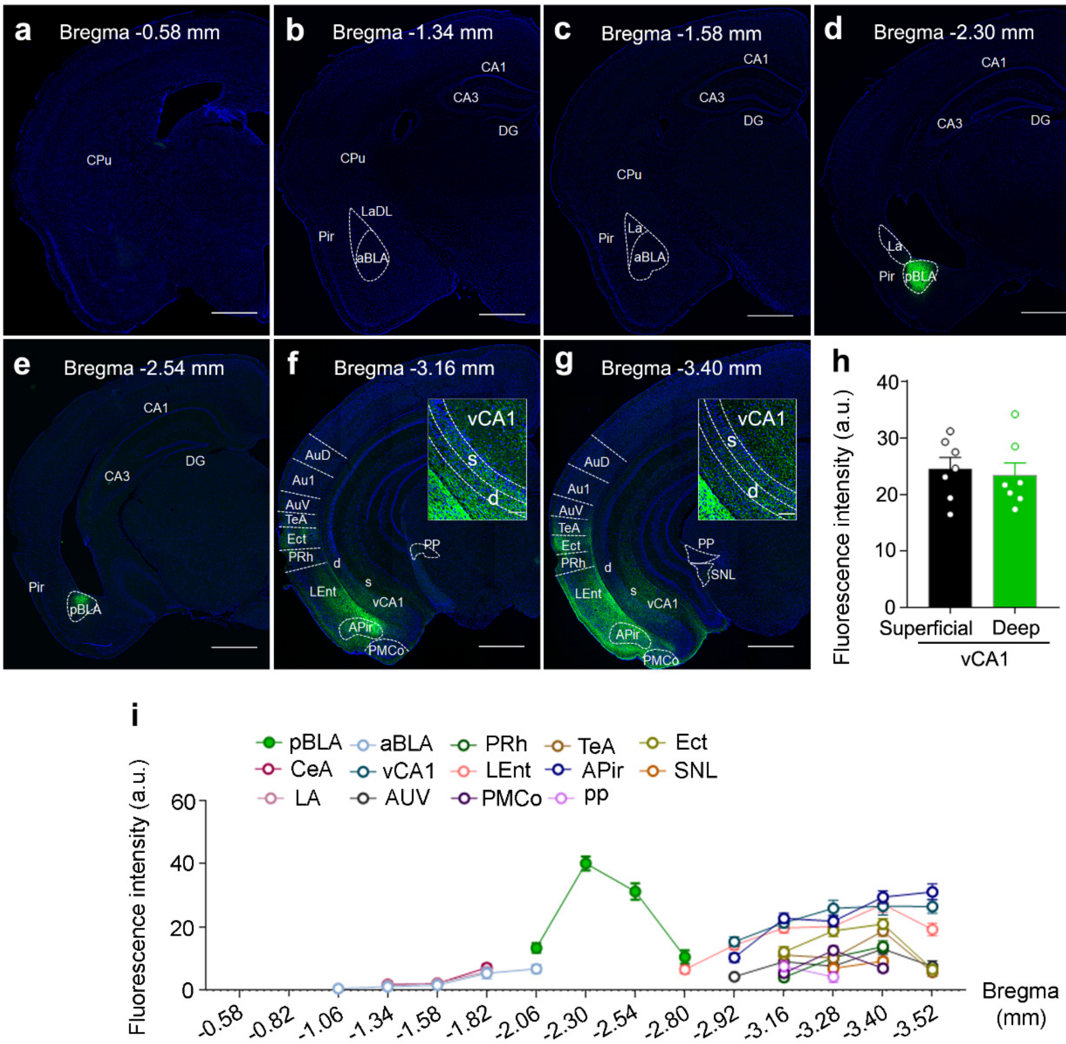

Supplementary Figure 7. Anatomical characterization of projections arising from pBLA. (a-g) Representative image showing ChR2-eYFP expression (green) localized to pBLA and the projections arising from the pBLA. **(h, i)** Quantitative analysis of the fluorescence intensity in pBLA (injection site) and its projection area (**i**), especially in the superficial and deep layer of the vCA1 (**h**). n=7 mice per group, paired t test, t=0.4737, df=6, P=0.6524. Data are presented as the mean ± SEM. CPu, caudate putamen; Pir, piriform cortex; La, lateral amygdaloid nucleus; Au1, primary auditory cortex; AuD, dorsal area of secondary auditory cortex; AuV, ventral area of secondary auditory cortex; TeA, temporal association cortex; Ect, ectorhinal cortex; PRh, perirhinal cortex; Lent, lateral entorhinal cortex; APir, amygdalopiriform transition area; PMCo, posteromedial cortical amygdaloid nucleus; PIL, posterior intralaminar thalamic nucleus; PP, peripeduncular nucleus; SNL, lateral part of substantia nigra; s, superficial; d, deep. Scale bar=1 mm (insets=100µm). Source data are provided as a Source Data file.

Supplementary Figure 8

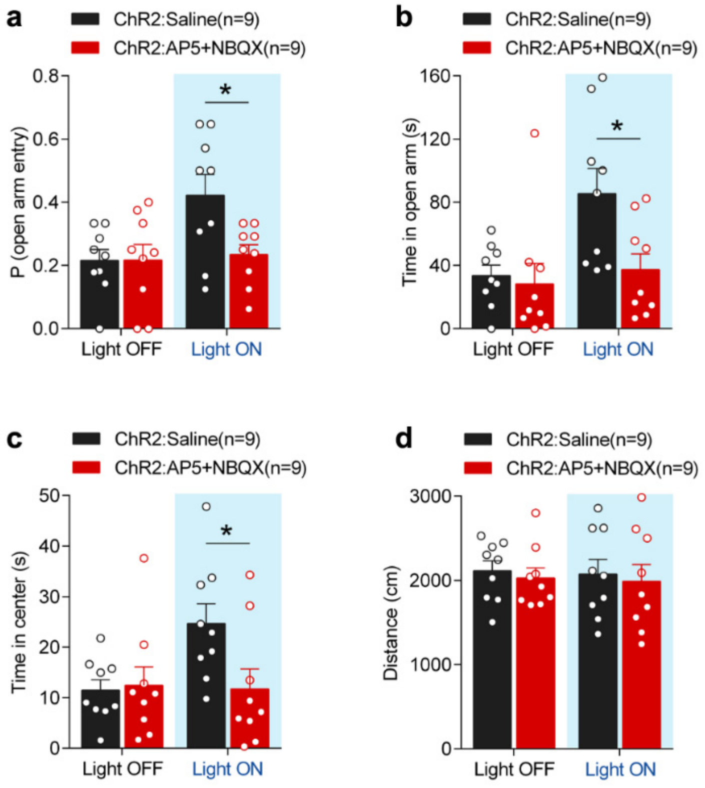

Supplementary Figure 8. Glutamatergic pBLA-vCA1 inputs mediate changes in anxiety-related behaviors.

Thirty min before behavioral assays (EPM and OFT) and photostimulation on pBLA-vCA1 terminals, GluR antag (AP5+NBQX) or saline as control were unilaterally infused locally into the vCA1 of ChR2-mice. (**a, b**) GluR antag blocked the light-induced increase of open arm entry probability (two-way ANOVA group × epoch interaction, $F_{(1, 16)} = 5.335$, $P = 0.0346$, Bonferroni post hoc, $*P < 0.05$) and time in the open arm (two-way ANOVA group × epoch interaction, $F_{(1, 16)} = 9.352$, $P= 0.0075$, Bonferroni post hoc, $*P < 0.05$). (**c**) After unilateral intra-vCA1 glutamate receptor blockade, photoactivation of ChR2-expressing pBLA terminals in the vCA1 failed to increase center exploration as seen after saline-treatment trials (two-way ANOVA group × epoch interaction, $F_{(1, 16)} = 9.075$, $P = 0.0083$, Bonferroni post hoc, $*P < 0.05$). (**d**) No effect of light stimulation or GluR antag injection was detected on the traveled distance (two-way ANOVA group × epoch interaction, $F_{(1, 16)} = 3.05e-005$, $P= 0.9957$). n=9 mice per group. Data are presented as mean ± SEM. Source data are provided as a Source Data file.

Supplementary Figure 9

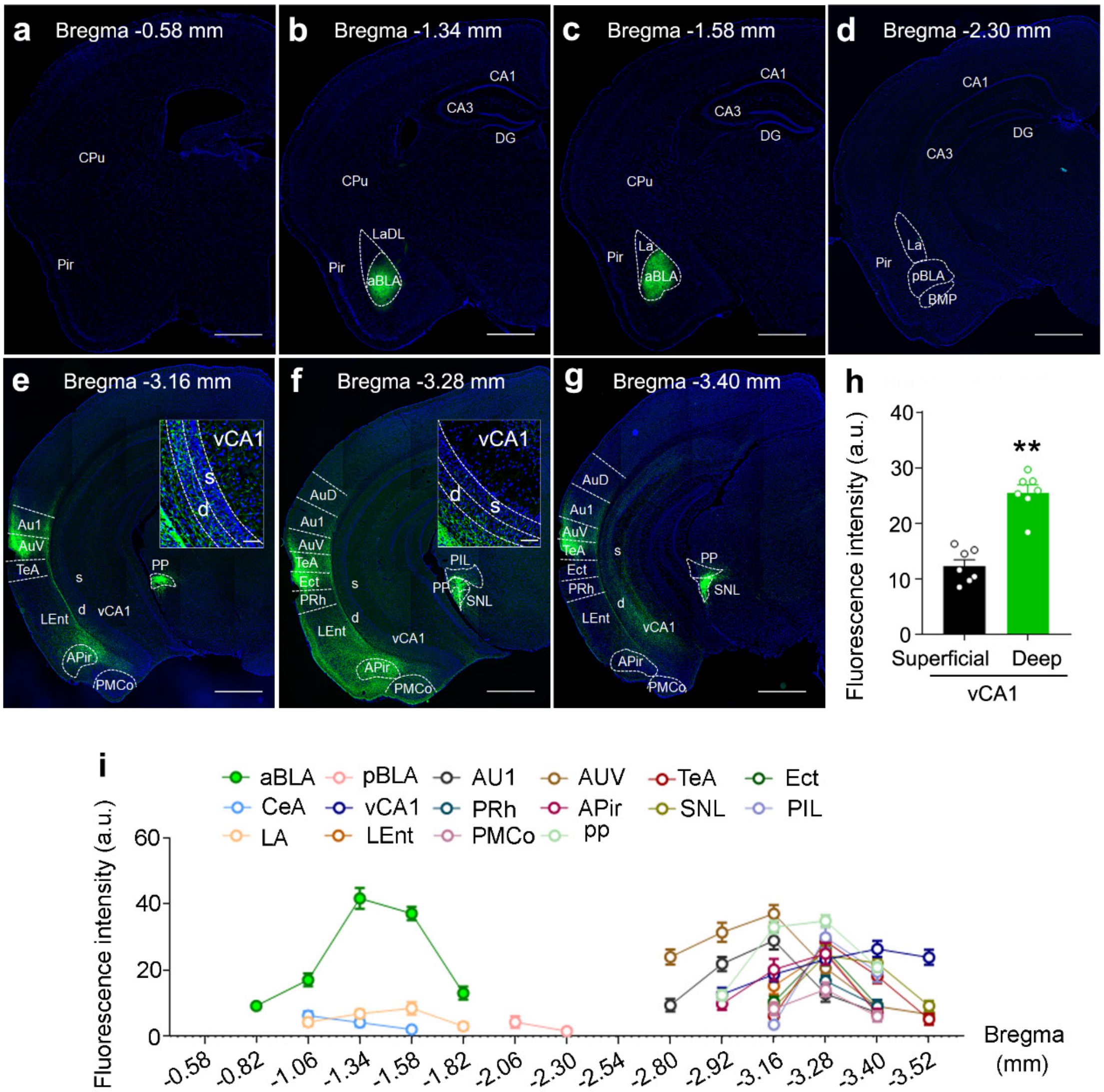

Supplementary Figure 9. Anatomical characterization of projections arising from aBLA. (a-g) Representative image showing ChR2-eYFP expression (green) localized to aBLA and the projections arising from the aBLA. **(i, h)** Quantitative analysis of the fluorescence intensity in aBLA (injection site) and its projection area (**i**), especially in the superficial and deep layer of the vCA1 (**h**). n=7 mice per group, paired t test, t=7.261, df=6, P=0.0003. Data are presented as the mean ± SEM. Scale bar=1 mm (insets=100μm). Source data are provided as a Source Data file.

Supplementary Figure 10

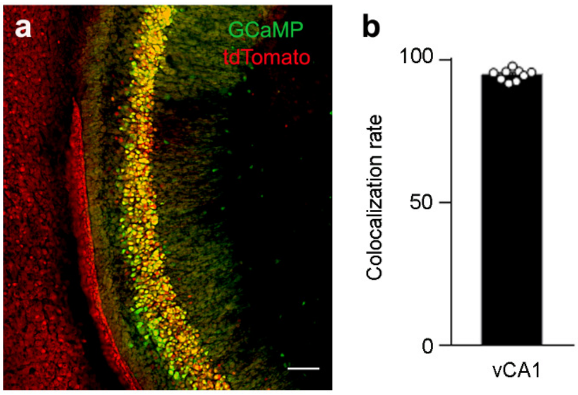

Supplementary Figure 10. Characterization of vCA1^{Calb1}–GCaMP mice.

(**a**) In Calb1-IRES2-Cre-D::Ai9 mice, AAV-EF1a-DIO-GCaMP6f virus was injected into the vCA1. Representative image of the injection site. Exogenously expressed GCaMP6f (green) was colocalized with Calb1 (tdTomato) in the vCA1. (**b**) Quantitative analysis of colocalization rate (yellow/green) in the vCA1 of vCA1^{Calb1}-GCaMP mice. Scale bar=100 µm. *n*=9 mice. Source data are provided as a Source Data file.

Supplementary Figure 11

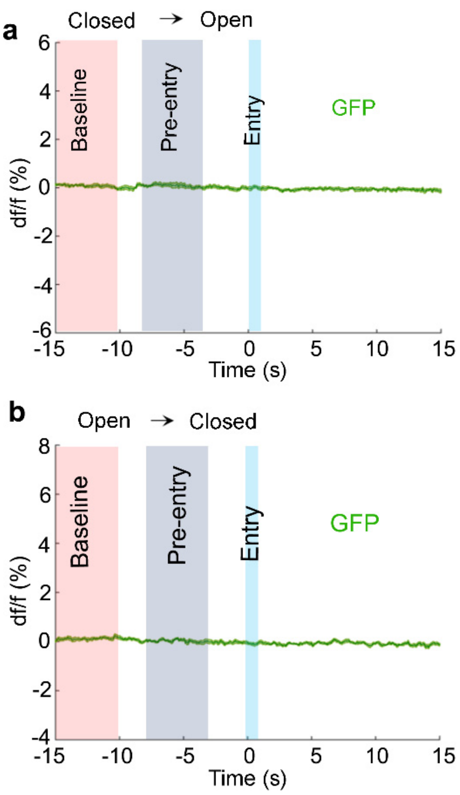

Supplementary Figure 11. No significant fluctuation of photometry in vCA1^{Calb1+}-eGFP neurons.

(**a**) Average photometry recordings from vCA1^{Calb1+}-neurons in the eGFP-mice during behavioral transition from the closed to open arm (transition point at time 0 s). (**b**) Same data format as (**a**), but the mice were moving from open to closed arm. *n*=8 mice per group. Source data are provided as a Source Data file.

Supplementary Figure 12

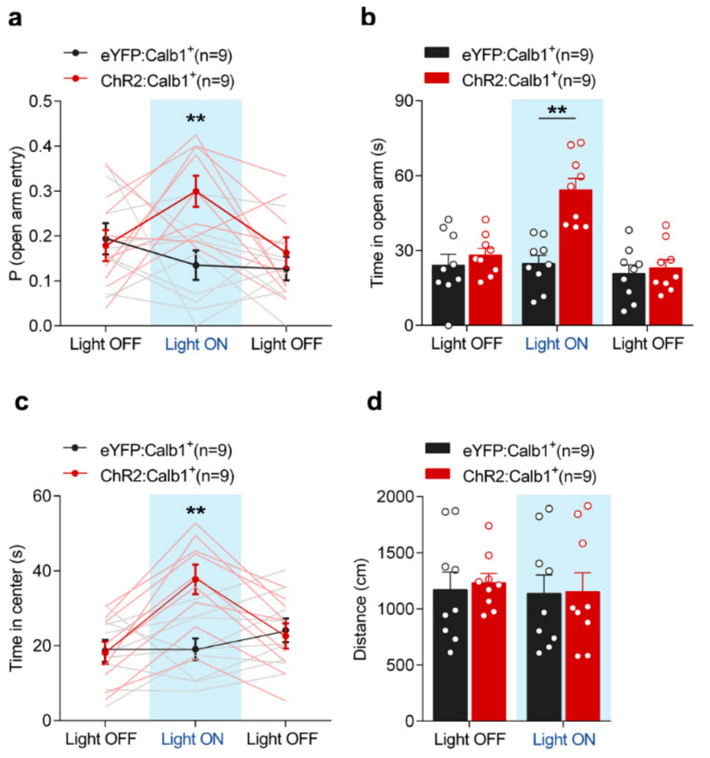

Supplementary Figure 12. Activation of vCA1^{Calb1+}-neurons decreases anxiety-related behaviors.

A single 9 min session in elevated plus maze (EPM) and open field test (OFT) was performed as described in Figure 2 **h-j**. (**a**) In EPM, optogenetic activation of vCA1^{Calb1+}-neurons increased probability of open-arm entry in ChR2 mice (two-way ANOVA group × epoch interaction, $F_{(2,\ 32)}$ = 4.3, P=0.0222; Bonferroni post hoc analysis, $**P < 0.01$). (**b**) During illumination epoch, time in the open arms was significantly increased in Calb1-ChR2 mice (two-way ANOVA group × epoch interaction, $F_{(2,\ 32)}$ = 16.5, P= 0.0001; Bonferroni post hoc analysis, $**P< 0.01$). (**c**) In OFT, Calb1-ChR2 mice spent significantly more time in the centre (two-way ANOVA group × epoch interaction, $F_{(2,\ 32)}$ = 22.07, P<0.0001; Bonferroni post hoc analysis, $**P< 0.01$) during the illumination epoch. (**d**) No effect of light stimulation was detected on the traveled distance (two-way ANOVA group × epoch interaction, $F_{(1,\ 16)}$ = 0.03607, P=0.8518). n=9 mice per group. Data are presented as mean ± SEM. Source data are provided as a Source Data file.

Supplementary Figure 13

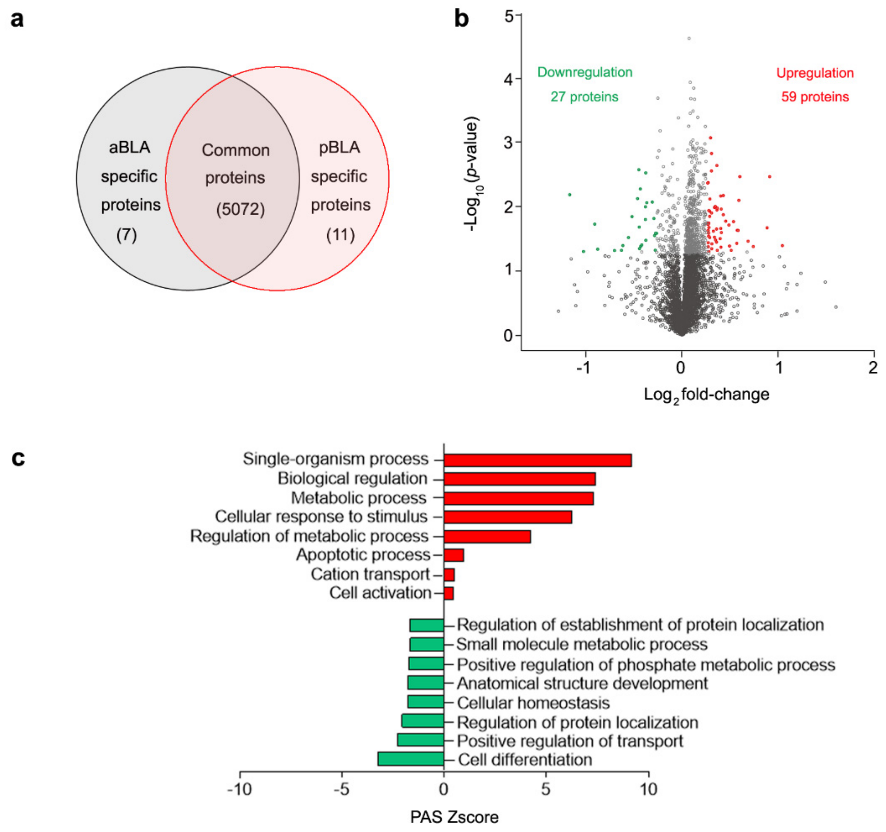

Supplementary Figure 13. Comparison of aBLA and pBLA expression proteomics in physiological condition.

(**a**) Venn diagram representing the number of proteins identified in the aBLA and pBLA of wild-type (Wt) mice. Of 5098 proteins identified, 5072 were grouped as differentially expressed proteins, 7 proteins were identified only in the aBLA and 11 were identified only in the pBLA. (**b**) Volcano plot showing the protein changes in the pBLA versus those in the aBLA of Wt mice. The *x* axis specifies the $\log_2$ fold change, and the *y* axis represents the negative $\log_{10}$ of the *p* values. Red and green dots represent proteins whose abundance was significantly increased or decreased in the pBLA versus aBLA (filtering criteria: FC > 1.2 or <0.833 and *P* value < 0.05). (**c**) Gene ontology (GO) enrichment analysis of up- and down- regulated GO categories in the pBLA versus aBLA. PAS Zscore is the pathway activation strength value, served as the activation profiles of the signaling pathways based on the expression of individual genes. *n*=3 mice per group.

Supplementary Figure 14

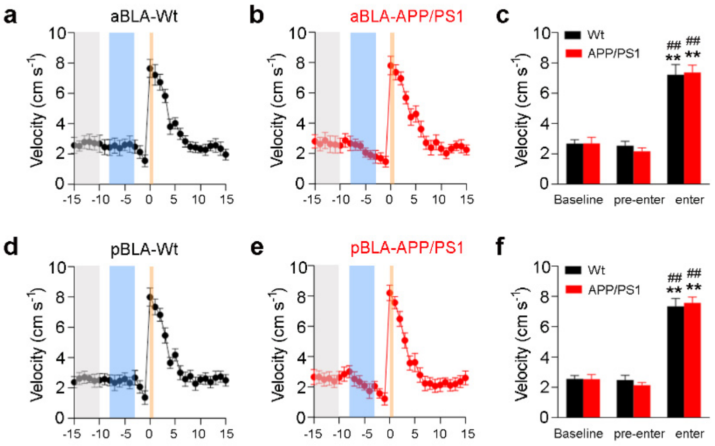

Supplemental Figure 14. Velocity of movements during photometry experiments in mice.

(a-d) Velocity of Wt **(a, d)** and APP/PS1 **(b, e)** mice from the closed arm to open arm in EPM. **(c, f)** Average velocity during baseline, pre-entry, and entry periods from **a, b** and **d, e**. Two-way RM ANOVA for Wt and APP/PS1 respectively, **P<0.01 vs. baseline; ## P<0.01 vs. pre-entry (**c**, **f**). Two-way RM ANOVA for Wt vs. APP/PS1, aBLA: $F_{(2, 36)}$ = 2.388, P=0.1062; pBLA: $F_{(2, 40)}$ = 3.059, P=0.0581. n=10 or 11 mice per group. Data are presented as mean ± SEM. Source data are provided as a Source Data file.

Supplementary Figure 15

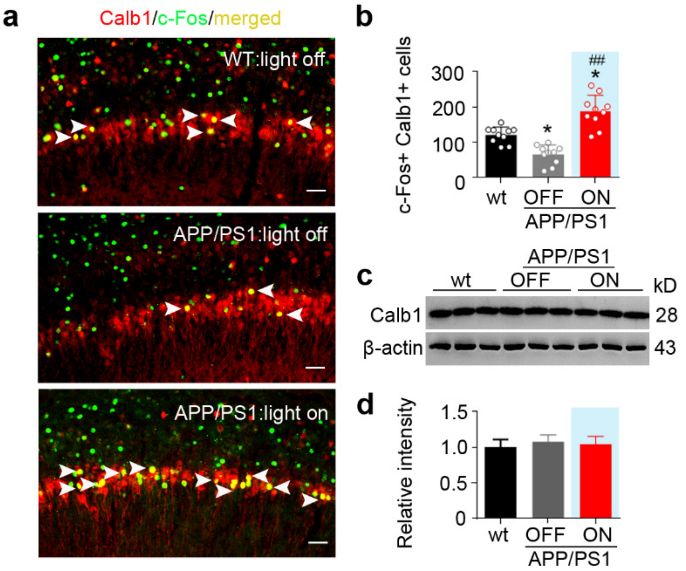

Supplementary Figure 15. Optostimulation rescues pBLA–vCA1 circuit without changing Calb1 level in AD mice.

(**a**) Representative co-staining of Calb1+/c-Fos+ neurons in the vCA1 of wild-type (Wt) and APP/PS1 mice after photoactivation of pBLA–vCA1 terminals. Scale bar, 50 µm. (**b**) The number of Calb1+/c-Fos+ neurons in the vCA1 of APP/PS1 mice significantly decreased compared with Wt mice, and photoactivation of pBLA–vCA1 inputs remarkably increased co-localization of Calb1+ (red) and c-Fos+ (green) neurons in the vCA1 of APP/PS1 mice (one-way ANOVA, n=10 per group, $F_{(2, 27)}$ = 33.77, P<0.0001, Tukey's multiple comparisons test, *P<0.05 vs. Wt, ## P<0.01 vs. OFF). (**c**) Western blotting to measure the protein level of Calb1. (**d**) Quantitative analysis of the protein level of Calb1 in the vCA1.n=3 mice per group. Source data are provided as a Source Data file.

Supplementary Figure 16

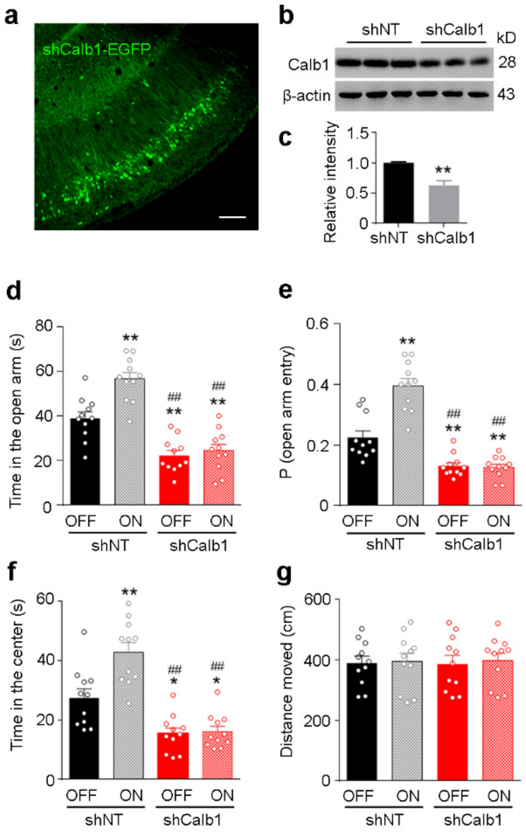

Supplementary Figure 16 Downregulating Calb1 in the vCA1 induces anxiogenic effects in mice.

(**a**) Representative image of the injection site in vCA1. Scale bar, 100 µm. (**b, c**) Expression of Calb1 was significantly decreased after AAV-ShCalb1 injection compared with AAV-ShNT group by Western blotting. *n*=3 per group. Unpaired *t* test, *t*=8.338, *df*=4, *P*=0.0011. (**d, e**) In EPM, shCalb1 mice showed less time staying in open arm (**d**) and probability of open-arm entry (**e**) compared with shNT mice. Optogenetic activation of pBLA–vCA1 inputs increased the time (**d**) and the open-arm entry (**e**) in shNT mice but not in shCalb1 mice (One-way ANOVA, time in open arm: $F_{(3, 40)}$=32.86, *P*<0.0001; probability of open arm entry: $F_{(3, 40)}$= 48.09, *P*<0.0001. (**f**) In OFT, shCalb1 mice showed significantly reduced staying time in the centre field (**f**) as compared with shNT mice. Optogenetic activation of pBLA–vCA1 inputs increased the time (**f**) in shNT mice but not in shCalb1 mice. One-way ANOVA, $F_{(3, 40)}$ = 25.08, *P*<0.0001. (**g**) Distance moved in OFT was not changed among the groups. One-way ANOVA, $F_{(3, 40)}$ = 0.03032, *P*=0.9928. *n*=11 mice per group, **P* < 0.05, ***P* < 0.01 vs. shNT OFF group, ##*P* < 0.01 vs. shNT ON group. Data are presented as mean ± SEM. Source data are provided as a Source Data file.

Supplementary Figure 17

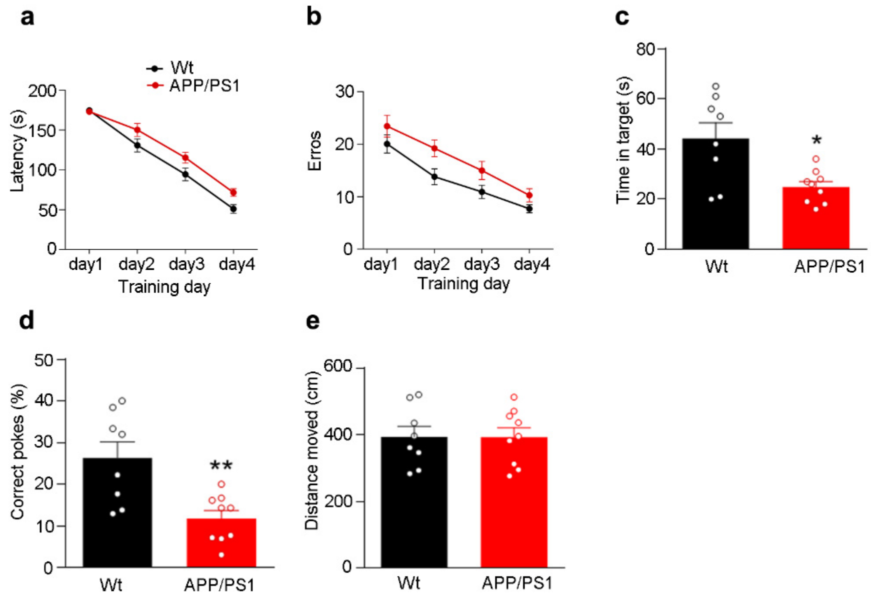

Supplementary Figure 17. Spatial memory deficit in APP/PS1 mice.

(**a, b**) APP/PS1 mice (6 Month-old) showed decreased latency and errors to find the correct pokes during Barnes maze (BM) training. (two-way ANOVA group × days, Latency: $F_{(3, 45)}$ = 1.545, P=0.2160; errors: $F_{(3, 45)}$ = 0.3101, P=0.8179). (**c**) In probe trial, APP/PS1 mice spent less time in the target quadrant (unpaired t test, t=2.962 df=8.735, P= 0.0164), with (**d**) decreased correct pokes (unpaired t test, t=3.352 df=10.26, P= 0.0071) compared with Wt mice. (**e**) No difference on the distance moved in BM was shown between two groups (unpaired t test, t=0.008496 df=14.41, P=0.9933). n=8 or 9 mice per group. Data are presented as mean ± SEM. Source data are provided as a Source Data file.
