# Supplementary material for: Posterior basolateral amygdala to ventral hippocampal CA1 drives approach behaviour to exert an anxiolytic effect
Source: Nat Commun. 2020 Jan 10;11:183. doi: 10.1038/s41467-019-13919-3 (PMC6954243; doi:10.1038/s41467-019-13919-3)
Supplement: Supplementary file 3 — Reporting Summary [file 41467_2019_13919_MOESM3_ESM.pdf]

nature research

Corresponding author(s): Jian-Zhi Wang

Last updated by author(s): Nov 22, 2019

Reporting Summary

Nature Research wishes to improve the reproducibility of the work that we publish. This form provides structure for consistency and transparency in reporting. For further information on Nature Research policies, see Authors & Referees and the Editorial Policy Checklist.

Statistics

For all statistical analyses, confirm that the following items are present in the figure legend, table legend, main text, or Methods section.

n/a	Confirmed	
☐	☒	The exact sample size (*n*) for each experimental group/condition, given as a discrete number and unit of measurement
☐	☒	A statement on whether measurements were taken from distinct samples or whether the same sample was measured repeatedly
☐	☒	The statistical test(s) used AND whether they are one- or two-sided<br>*Only common tests should be described solely by name; describe more complex techniques in the Methods section.*
☐	☒	A description of all covariates tested
☐	☒	A description of any assumptions or corrections, such as tests of normality and adjustment for multiple comparisons
☐	☒	A full description of the statistical parameters including central tendency (e.g. means) or other basic estimates (e.g. regression coefficient) AND variation (e.g. standard deviation) or associated estimates of uncertainty (e.g. confidence intervals)
☐	☒	For null hypothesis testing, the test statistic (e.g. *F*, *t*, *r*) with confidence intervals, effect sizes, degrees of freedom and *P* value noted<br>*Give P values as exact values whenever suitable.*
☒	☐	For Bayesian analysis, information on the choice of priors and Markov chain Monte Carlo settings
☒	☐	For hierarchical and complex designs, identification of the appropriate level for tests and full reporting of outcomes
☐	☒	Estimates of effect sizes (e.g. Cohen's *d*, Pearson's *r*), indicating how they were calculated

Our web collection on statistics for biologists contains articles on many of the points above.

Software and code

Policy information about availability of computer code

Data collection	Data was collected using optical fiber recording system(QAXK-FPS-MC, Thinker Tech Nanjing Biotech Limited Co., Ltd), video–tracking and behavioral analysis software(TMV-100S) . Electrophysiological recordings were collected using Clampfit software.
Data analysis	Data was analyzed using GraphPad Prism version 7, Image J 64 and Matlab. Analyses of bioinformatics data were carried out with Perseus software, Microsoft Excel and R statistical computing software.

For manuscripts utilizing custom algorithms or software that are central to the research but not yet described in published literature, software must be made available to editors/reviewers. We strongly encourage code deposition in a community repository (e.g. GitHub). See the Nature Research guidelines for submitting code & software for further information.

Data

Policy information about availability of data

All manuscripts must include a data availability statement. This statement should provide the following information, where applicable:

- Accession codes, unique identifiers, or web links for publicly available datasets
- A list of figures that have associated raw data
- A description of any restrictions on data availability

Source data for all graphs in the figures of this manuscript are provided in Source Data file.

Field-specific reporting

Please select the one below that is the best fit for your research. If you are not sure, read the appropriate sections before making your selection.

☒ Life sciences ☐ Behavioural & social sciences ☐ Ecological, evolutionary & environmental sciences

Life sciences study design

All studies must disclose on these points even when the disclosure is negative.

Sample size	Power analysis was not used, instead sample sizes were based upon previously published variability of assays and sufficient numbers for duplication. The sample sizes are clearly indicated in the figure legends.
Data exclusions	Animals were excluded from behavioral assays based on incorrect targeting with virus or incorrect placement of cannula.
Replication	Behavioral assays were repeated a minimum of 2 times with successful replication of results.
Randomization	Mice were randomly assigned to experimental groups.
Blinding	The investigators were blind to the experimental conditions.

Reporting for specific materials, systems and methods

We require information from authors about some types of materials, experimental systems and methods used in many studies. Here, indicate whether each material, system or method listed is relevant to your study. If you are not sure if a list item applies to your research, read the appropriate section before selecting a response.

Materials & experimental systems

n/a	Involved in the study	
☐	☒	Antibodies
☒	☐	Eukaryotic cell lines
☒	☐	Palaeontology
☐	☒	Animals and other organisms
☒	☐	Human research participants
☒	☐	Clinical data

Methods

n/a	Involved in the study	
☒	☐	ChIP-seq
☒	☐	Flow cytometry
☒	☐	MRI-based neuroimaging

Antibodies

Antibodies used	Primary antibodies were against Calbindin (Rabbit ,1:1000/1:300;abcam ab11426),β–Actin (Rabbit,1:5000;abclonal AC026), C-fos (Goat,1:200; Santa Cruz sc-52-g),monoclonal mouse 6E10 (1:300; 803001, biolegend) .<br>Secondary antibodies were conjugated to Alexa Fluor 488 (donkey to rabbit IgG(H+L) ,1:300, Invitrogen A21206; donkey to mouse IgG(H+L) ,1:300; Invitrogen A21202) and odessey(800) (goat anti rabbit ,1:10000; biosciences 926-32211).
Validation	These are usually used antibodies and evidence for the validation, including some references can be found on manufacturer's website.

Animals and other organisms

Policy information about studies involving animals; ARRIVE guidelines recommended for reporting animal research

Laboratory animals	APP/PS1 mice: Model Animal Research Center of Nanjing University (Nanjing, China), NO.D000268<br>Calb1–IRES2–Cre–D(B6.Cg–Calb1tm2.1(cre)Hze/J: Jackson Laboratory (USA), No.028532<br>tdTomato reporter Ai9 mice: Jackson Laboratory (USA), No. 007905<br>Adult male C57BL/6 mice (p45–60): Beijing Vital River Laboratory Animal Technology Co., Ltd.
Wild animals	*Provide details on animals observed in or captured in the field; report species, sex and age where possible. Describe how animals were caught and transported and what happened to captive animals after the study (if killed, explain why and describe method; if released, say where and when) OR state that the study did not involve wild animals.*
Field-collected samples	The study did not involve samples that were collected from the field.
Ethics oversight	Animal Care and Use Committee of Huazhong University of Science and Technology

Note that full information on the approval of the study protocol must also be provided in the manuscript.
